# A *PIK3R2* Mutation in Familial Temporal Lobe Epilepsy as a Possible Pathogenic Variant

**DOI:** 10.3389/fgene.2021.596709

**Published:** 2021-05-10

**Authors:** Yishu Wang, Jing Peng, Shuwei Bai, Haojun Yu, Hong He, Chunxiang Fan, Yong Hao, Yangtai Guan

**Affiliations:** ^1^Department of Neurology, Renji Hospital, Shanghai Jiao Tong University School of Medicine, Shanghai, China; ^2^Department of Neurology, The Second Affiliated Hospital of Xinjiang Medical University, Ürümqi, China; ^3^School of Medical Instrument and Food Engineering, University of Shanghai for Science and Technology, Shanghai, China; ^4^TCM Department, Shanghai Punan Hospital of Pudong New District, Shanghai, China; ^5^Department of Neurology, Ningbo Hangzhou Bay Hospital, Ningbo, China

**Keywords:** *PIK3R2*, temporal lobe epilepsy, familial temporal lobe epilepsy, genetic epilepsy, iPSCs

## Abstract

Temporal lobe epilepsy (TLE), the most common form of medically refractory focal epilepsy in adults, often requires surgery to alleviate seizures. By using next-generation sequencing, we identified a *PIK3R2* mutation (NM_005027.4: c.265C > T; NP_005018.2: p.Arg89Cys) in a family with mesial temporal lobe epilepsy. *PIK3R2* encodes p85β, the regulatory subunit of Class IA phosphoinositide 3-kinase (PI3K) and the mutation we identified in *PIK3R2* seems to function unexpectedly as a possible pathogenic variant. The mutation is predicted to be potentially pathogenic by multiple bioinformatics tools. Through a functional assay, we verified that the mutation enhances the function of PI3K in induced pluripotent stem cells (iPSCs) derived from peripheral blood mononuclear cells (PBMCs) of the proband. Finally, pathological testing of the resected temporal lobe cortex showed that the expression of *PIK3R2* was significantly higher in patients with refractory temporal lobe epilepsy than in those of non-epileptic diseases as a control group. It can be inferred that *PIK3R2* might play an important role in the development of TLE.

## Introduction

Temporal lobe epilepsy (TLE), the most common type of refractory focal epilepsy in adults, often requires surgery to resolve the associated seizures. TLE is mainly divided into two subtypes: mesial temporal lobe epilepsy (mTLE) and lateral temporal lobe epilepsy (lTLE) (Allone et al., 2017). mTLE seizures generally originate from the hippocampus, parahippocampal gyrus, and amygdala. mTLE is characterized by prominent auras (rising epigastric auras, déjà vu, or a sense of strangeness, etc.), mainly caused by hippocampal sclerosis (HS) (Asadi-Pooya et al., 2016; Allone et al., 2017). Familial mesial temporal lobe epilepsy (FMTLE), first recognized by Berkovic et al. (1996) was originally described as a benign syndrome with prominent déjà vu. Most of the seizure types are simple partial seizures, rare complex partial seizures, and rarer secondary generalized seizures. As more mTLE pedigrees are reported, the complexity of FMTLE is being recognized. The severity of FMTLE in different families varies from benign epilepsy syndrome to drug-resistant refractory epilepsy with a variety of auras and manifestations, including psychic symptoms, automatism, déjà vu, rising epigastric or nausea, sensory abnormalities, and fear (Cendes et al., 1998; Crompton et al., 2010; Perucca et al., 2017). In particular, magnetic resonance imaging (MRI) of some patients has revealed hippocampal sclerosis involving hippocampal atrophy and an increased T2WI signal (Kobayashi et al., 2001).

In terms of the genetic model, some FMTLE families show autosomal dominant inheritance and incomplete dominance, whereas a few exhibit recessive inheritance (Crompton et al., 2010). Because of the complex inheritance patterns, the genetic pathogenesis of FMTLE remains largely elusive. Nonetheless, several loci have been associated with FMTLE, including 4q13.2-q21.3, 3q25-q26, 18qter, 1q25-31, and 12q22-23.3 (Baulac et al., 2001; Claes et al., 2004; Hedera et al., 2007; Chahine et al., 2013; Fanciulli et al., 2014), but the pathogenic gene responsible for FMTLE is still unknown.

Class IA phosphoinositide 3-kinase (PI3K) enzymes and related signaling pathways, e.g., PI3K/Akt/mTOR, play a wide range of pathophysiological roles in cell proliferation, growth, metabolism, migration, and secretion (Fruman et al., 2017; Dornan and Burke, 2018; Hillmann and Fabbro, 2019; Vallejo-Diaz et al., 2019). The PI3K/Akt/mTOR pathway is crucial in neuronal development and immigration. Germline or somatic mutations in genes regulating the PI3K/Akt/mTOR pathway can cause focal cortical dysplasias (FCDs), which are malformations of cortical development (MCDs) that are highly associated with drug-resistant refractory epilepsy and are the most common cause of neocortical epilepsy in children (Iffland and Crino, 2017). The *PIK3R2* gene on chromosome 19p13.11 encodes the p85β regulatory subunit of Class IA PI3K, which is important for the activation of PI3K. It has been reported by several studies that multiple *PIK3R2* mutations are the driving factors of two epilepsy-related developmental brain disorders, megalencephaly-polymicrogyria-polydactyly-hydrocephalus syndrome (MPPH) and bilateral perisylvian polymicrogyria (BPP). Riviere et al. (2012) identified a *de novo* heterozygous germline mutation in *PIK3R2* (c.1117G > A; p.Gly373Arg) in 13 patients with MPPH and confirmed by functional assays that the mutation significantly increased the activity of PI3K. Thereafter, Nakamura et al. (2014) reported a *de novo* heterozygous missense mutation at another site of *PIK3R2* (c.1202T > C, p.Leu401Pro) in a case of MPPH, but it was not functionally verified. In addition to MPPH, mutation of *PIK3R2* (c.1117G > A; p.Gly373Arg) has also been detected in 19 patients with BPP, and a nearby missense mutation (1126A > G; Lys376Glu) was identified in one patient, as reported by Mirzaa et al. (2015). Another *de novo* mutation at a new site of *PIK3R2* (c.1669G > C; p.Asp557His) was reported in a patient with MPPH by Terrone et al. (2016) without functional verification. Although an increasing number of mutations in *PIK3R2* have been identified in patients with epilepsy-related diseases, their influence is rarely revealed by functional assays.

Based on existing findings, mutations in *PIK3R2* can cause excessive activation of PI3K/Akt/mTOR, resulting in overgrowth of neurons to form developmental brain disorder syndrome, both MPPH and BPP, as characterized by seizure and other clinical features. However, *PIK3R2* mutations have not been reported in non-MPPH or BPP syndromes related to epilepsy thus far. In this article, we describe a mutation in *PIK3R2* (NM_005027.4: c.265C > T; NP_005018.2: p.Arg89Cys) identified in an FMTLE family. It is the first time for this mutation to be reported as a potential pathogenic variant. The bioinformatics analysis suggests that this heterozygous missense mutation is pathogenic. A functional assay using induced pluripotent stem cells (iPSCs) generated from peripheral blood mononuclear cells (PBMCs) of the proband confirmed that PI3K activity was upregulated in the cells as a result of the *PIK3R2* mutation. Expression of *PIK3R2* in the resected brain tissue of patients with refractory temporal lobe epilepsy was also significantly higher than that in a non-epileptic control group.

## Materials and Methods

### Subjects

The proband and her youngest sister were treated in the Department of Neurology of Renji Hospital Affiliated with Shanghai Jiao Tong University School of Medicine. The proband and her biological family members signed informed consent forms for inclusion before they participated in the study. The study was conducted in accordance with the Declaration of Helsinki, and the protocol was approved by the Medical Ethics Committee of Renji Hospital Affiliated with Shanghai Jiao Tong University School of Medicine (RA-2019-160).

### Samples

A 4-ml peripheral blood sample of the proband and her family members was collected with EDTA anticoagulant. Next-generation sequencing of a panel containing 511 epilepsy-related genes (see Supplementary Table 1) and Sanger sequencing for the family members were carried out at Beijing Kangso Medical Inspection.

### Genomic DNA Extraction and Amplification

Genomic DNA was extracted from blood samples using the Qiagen FlexiGene DNA kit (Qiagen, Germany) according to the manufacturer’s instructions and was stored at −80°C. The PCR procedure was as follows: 95°C for 10 min, 35 cycles (95°C for 30 s, 60°C for 30 s, 72°C for 45 s) followed by a final extension step at 72°C for 5 min.

### Selection of Target Genes and Design of Probes

Five hundred and eleven epilepsy-related genes were selected as target genes based on information from the HGMD and OMIM databases. Capture probes were designed for the sequencing of the exons and intronic flanking sequences (±10 bp) of the target genes using the Agilent SureDesign (Agilent, United States) online design tool.

### DNA Library Construction

The DNA library was constructed by using Sure Select Target Enrichment System (Agilent, United States) for the enrichment of target sequences according to the manufacturer’s instructions. The genomic DNA sample was fragmented into 100∼500-bp DNA fragments by an ultrasonic processor. Adaptors were ligated to both ends of the DNA fragments, and cohesive ends were trimmed. The DNA library was amplified and purified by PCR.

### Hybridization, Capture, and Amplification

The target DNA fragments of the amplified DNA library were hybridized and captured by probes using SureSelectXT Reagent Kit (Agilent, United States) and streptavidin magnetic beads and amplified by PCR. Then, the products were purified and quantified.

### Sequencing and Data Analysis

Next-generation sequencing was performed using a NEXTSEQ500 sequencer (Illumina, United States). The original data were analyzed by real-time analysis (RTA, Illumina), the Burrows–Wheeler Alignment Tool (BWA), and Genome Analysis Toolkit (GATK) bioinformatics software. The locations of variants in genomes and transcripts were annotated with ANNOVAR. The frequency of variants in the general population was annotated with 1,000 Genomes and dbSNP.

### Sanger Sequencing Validation

The gene sequences of variants were acquired from GenBank. The primers were designed and synthesized by Primer Z^1^. DNA samples from the proband and her biological family members were subjected to PCR to amplify variants, and then Sanger sequencing was performed. The results obtained were aligned with the previous results of next-generation sequencing, and false-positive sites obtained by NGS were ruled out.

### Protein Structure Modeling

The I-TASSER online server^2^ was used to examine the primary protein structure of p85β, and PyMOL molecular graphics software^3^ was utilized to predict the local structure within the area of p.Arg89Cys. The effects of the variants on protein function were predicted by PolyPhen-2 (Adzhubei et al., 2010), SIFT (Choi and Chan, 2015), Mutation Taster (Schwarz et al., 2014), and CADD (Rentzsch et al., 2021).

### Generation and Validation of the iPSC Line

Peripheral blood mononuclear cells from the proband were reprogrammed to generate an iPSC line as described previously (Zhang et al., 2020). Briefly, after confirming that the PBMCs carried the *PIK3R2* mutation, PBMCs were reprogrammed by the non-integrating vector system with human OKSM transcription factors, including Oct3/4, Klf4, Sox2, c-Myc using a CytoTune-iPS 2.0 Sendai Reprogramming Kit according to the manufacturer’s protocol. After 20 days of transduction, iPSC colonies were selected and passaged. From the 5th passage of iPSCs, the absence or presence of the vector was analyzed by PCR and gel electrophoresis. Elimination of the reprogramming factors was confirmed after the 7th passage. The karyotype of the iPSC line was inspected by G-band analysis, and the results showed a normal diploid 46, XX karyotype, without any detectable abnormality. Expression of the pluripotent markers Nanog, Sox2, SSEA4, TRA-1-60, and Oct3/4 was confirmed by flow cytometry and immunofluorescent staining. The differentiation capacity of the iPSCs into the three germ layers was demonstrated by in vitro expression of ectoderm (Pax6), mesoderm (Brachyury), and endoderm (AFP) markers. For the culture of the iPSC line, the culture plates were pre-coated with Matrigel (Corning, United States, 354230) for 1 h at 37°C prior to cell seeding. The iPSCs were cultured in iPSCs culture medium (HELP, China, HELP3110) and the culture medium was changed every 24 h. The iPSCs were passaged every 3–4 days and TrypLE (Gibco, United States, 12605010) was used for cell digestion.

### Construction of Protein–Protein Interaction (PPI) Network

We first searched the DisGeNET platform^4^ for TLE-related genes and obtained a gene set containing 354 components. Then the STRING database^5^ was used to find out the interaction between products of *PIK3R2* and the TLE-related genes. The information of protein–protein interactions of PIK3R2 was kept while the interactions which are not relevant to PIK3R2 were discarded. For the visualization of the results, Cytoscape software (version 3.5.1) was used to show the interaction between products of *PIK3R2* and 32 TLE-related genes in an image. The area of the circles representing 32 TLE-related genes is proportional to the combined score between *PIK3R2* and the TLE-related genes.

### Immunofluorescence

Induced pluripotent stem cells were grown on Matrigel (Corning, United States)-coated coverslips before subsequent staining. The cells were immobilized with 4% paraformaldehyde in PBS (15 min) and permeabilized with 0.25% Triton X-100 in PBS (10 min), followed by blocking with 1% BSA in PBST (PBS+ 0.1% Tween 20). Then, the cells were incubated with an anti-phosphatidylinositol-3,4,5-trisphosphate (PIP3) monoclonal antibody (1:50; MBL, Japan; D145-3) overnight at 4°C, followed by a goat anti-mouse IgG (Alexa Fluor® 594) (1:800; Abcam, United Kingdom; ab150116) secondary antibody for 1 h at room temperature. Finally, the cells were incubated with Hoechst stain (5 min) before mounting with mounting medium (Thermo Fisher Scientific, United States).

### Immunohistochemistry

Temporal lobe tissues were collected from the Department of Neurosurgery and Functional Neurology at Renji Hospital. We collected the temporal lobe tissues from 66 patients with refractory temporal lobe epilepsy and 40 non-epileptic patients with cerebral hemorrhage treated by surgical removal of the unilateral temporal lobe and hippocampus. Temporal lobe tissue blocks were fixed in 4% paraformaldehyde for 24 h and dehydrated in 30% sucrose solution, followed by embedding in paraffin. The tissues were dissected into 6-μm sections and then deparaffinized, rehydrated, and blocked with 2.5% normal donkey serum overnight at 4°C. The sections were incubated with an anti-PI3K p85β monoclonal antibody (1:100; Abcam, United Kingdom; ab180967) overnight at 4°C, followed by a biotinylated secondary antibody. The avidin-biotin-peroxidase complex and diaminobenzidine were used for visualization. Finally, the slides were counterstained with hematoxylin. ImageJ software was used to calculate the percentage of the positive area of three fields of each section.

### Statistical Analysis

Statistical analysis was performed using SPSS Software (Version 26). The Shapiro–Wilk test was used for normality analysis of data distribution. Two-tailed *t*-tests were used to compare the signal intensity of iPSCs of the proband and the healthy control. The Mann–Whitney test was employed to compare the percentage of the positive area of p85β in temporal lobe tissue sections of TLE and control patients. A *P*-value < 0.05 was considered statistically significant.

## Results

### Clinical History

**FIGURE 1:**
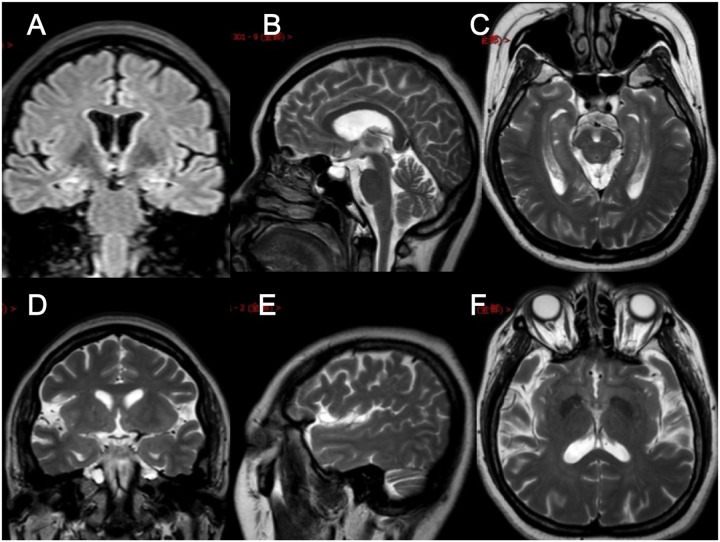
Brain MRI images of the proband. **(A–F)** Brain MRI images of different levels of the proband show hippocampal atrophy. **(A)** FLAIR in the coronal plane; **(B,E)** T2WI in the sagittal plane; **(C,F)** T2WI in the axial plane; **(D)** T2WI in the coronal plane.

In clinical work, we encountered a family of FMTLE. The proband was a 48-year-old female patient with a 5-year history of epilepsy at the time of her first visit. Her seizures were characterized by a rising epigastric aura followed by three types of seizures: absence for 2–3 s and then regain consciousness; automatism, including smacking her lips, groping, and swallowing; and secondary generalized seizure. Focal seizures occurred once to twice every week, and secondary generalized seizures occurred every 2–3 months. Her seizures could be induced by fatigue or mood swings but only occurred in the daytime. Cognitive function of the proband via evaluations showed that she had largely normal cognition, with a Mini-Mental State Examination (MMSE) score of 29 (normal value interval: 27–30) and a Montreal Cognitive Assessment (MoCA) score of 25 (normal value interval: 26–30). The proband had obvious anxiety and depression symptoms with a 9-item Patient Health Questionnaire (PHQ-9) score of 13 (normal value interval: 0–4) and a 7-item Generalized Anxiety Disorder questionnaire (GAD-7) score of 8 (normal value interval: 0–4). Specifically, hippocampal MRI of the proband revealed atrophy of the bilateral hippocampus, which is a characteristic manifestation of mTLE (Figure 1), while no obvious abnormality was found on the electroencephalogram (EEG). At present, the proband is being treated with sodium valproate (0.5 g, bid) and oxcarbazepine (0.6 g, bid). In addition, preoperative evaluation for refractory epilepsy surgical treatment was recommended, but the patient refused.

**FIGURE 2:**
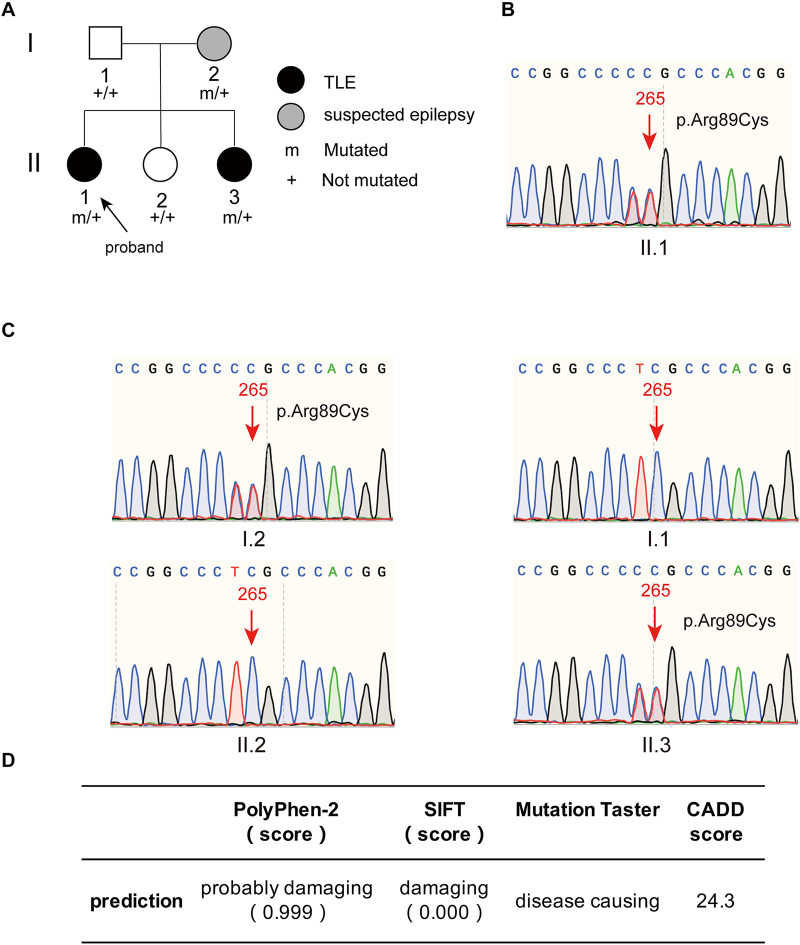
The family tree of the proband and the mutant site of *PIK3R2* identified in the patients. **(A)** The family tree of the proband shows that the mother is a suspected epilepsy patient, the proband and her second sister are TLE patients, while her father and first sister are not epilepsy patients. **(B,C)** The *PIK3R2* mutation (c.265 C > T; p.Arg89Cys) was detected in the proband, her mother, and her second sister, showing a heterozygous missense mutation. A variant c. 264 T > C was also detected, however, it was a synonymous variant and found to be an SNP site, which does not affect the function of the protein. **(D)** The influence of the mutation c.265 C > T (p.Arg89Cys) on the function of protein was predicted to be “probably damaging,” “damaging,” and “disease causing” by PolyPhen-2, SIFT, and Mutation Taster, separately; and the CADD score of this variant is also shown.

The proband had two younger sisters, and her youngest sister also had temporal lobe epilepsy. This patient was a 43-year-old female who experienced her first seizure at 40 years old. She had symptoms similar to those of the proband and experienced déjà vu auras prior to seizures. The frequency of her seizures was initially once a year and gradually became once every 3 months on average; at present, seizures are occurring once a month. The younger sister and father of the proband were healthy, without a history of epilepsy or febrile seizures. The proband’s mother had experienced a transient loss of consciousness in her young adulthood, with suspicion of epilepsy. The family tree with regard to epilepsy is depicted in Figure 2A.

### Genetic Findings

DNA extracted from the proband’s PBMCs was subjected to next-generation sequencing (NGS) targeting a panel of 511 selected genes involved in epilepsy (see Supplementary Table 1). Three mutations in *PIK3R2, FLNA*, and *KCNS3* were identified. The heterozygous missense variation c. 1582G > A (p.Val528Met) was found in the *FLNA* gene. Sanger sequencing confirmed that the proband’s mother and youngest sister carried the same heterozygous variation c.1582G > A (p.Val528Met); no mutation was found in the *FLNA* gene of her father and the other sister. However, diseases caused by *FLNA* gene mutation are X-linked recessive inherited, and the genetic model of the above family obviously does not support X-linked recessive inheritance; thus, the possibility of the disease caused by FLNA mutations was ruled out. Another heterozygous missense variation, c.722T > C (p.Leu241Pro), was found in the *KCNS3* gene. Sanger sequencing confirmed that except for the father of the proband, all family members harbored the same *KCNS3* mutation as the proband. Although the genetic mode of KCNS3 is not clear, the results of Sanger sequencing do not support the pathogenic role of *KCNS3* gene variation in this family.

A heterozygous missense mutation, c.265C > T (p.Arg89Cys), was found in the *PIK3R2* gene of the proband (II.1) and confirmed by Sanger sequencing (Figure 2B). Sanger sequencing also confirmed that the youngest sister (II.3) and mother (I.2) of the proband had the same heterozygous missense mutation, but neither the father (I.1) nor the younger sister (II.2) did (Figure 2C). It is necessary to explain that a heterozygous variant, c.264T > C was also detected in the proband, her mother, and her youngest sister, as shown in Figures 2B,C. This c. 264 T > C variant is a synonymous variant and also an SNP site, which is unlikely to be a pathogenic variant, so we did not study it further. The c.265C > T (p.Arg89Cys) variant has not been reported previously in available databases of pathogenic variants (HGMD Pro, ClinVar, PubMed, and GeneMatcher). It is a rare variant according to the mutation frequency presented in the 1,000 Genomes database. This variant may be pathogenic, as *PIK3R2* mutations have been reported to be the causes of MPPH and BPP, which can lead to epileptic seizures in patients in an autosomal dominant genetic pattern. The inheritance pattern of this *PIK3R2* mutation and the clinical phenotype of this family fits the pattern of genetic variants co-segregation. This mutation is predicted to be “probably damaging,” “damaging,” and “disease causing” through the online tools PolyPhen-2, SIFT, and Mutation Taster, respectively; and the CADD score of this mutation is 24.3, showing the potential deleteriousness (Figure 2D). We compared its CADD score with those of other pathogenic variants of *PIK3R2* recorded in the ClinVar database. As shown in Supplementary Table 2, seven pathogenic variants of *PIK3R2* have been identified in previous studies, and the CADD scores of them ranged from 23.5 to 30. It can be seen that the CADD score of the mutation reported here is within this range, implying its high pathogenic potential. Based on the above detection and analysis results, we speculate that *PIK3R2* c.265C > T (p.Arg89Cys) may be involved in the pathogenesis of FMTLE.

### Bioinformatics Analysis

#### Structural Modeling

**FIGURE 3:**
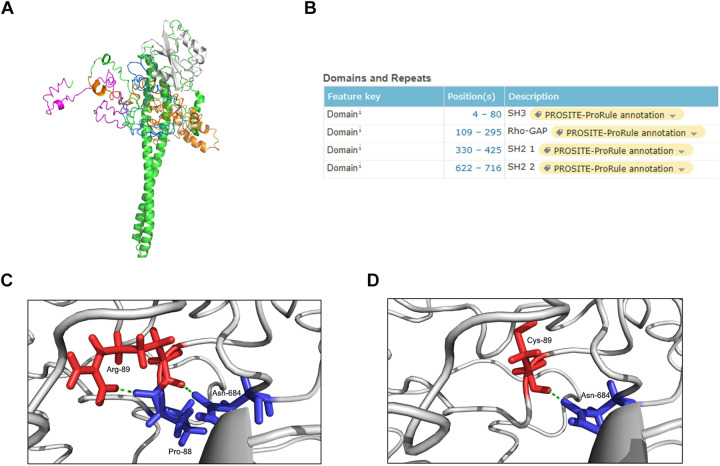
Structure modeling of the complete protein of *PIK3R2* and the local structure of the mutation site in wild-type and mutant protein. **(A)** The complete structure of the p85β subunit: SH3 domain (pink), Rho-Gap domain (orange), SH2 1 domain (white), SH2 2 domain (blue), and other regions (green). **(B)** The position information of four major domains on the UniProt Database. It shows that the mutation site c.265 C > T (p.Arg89Cys) locates near the SH3 domain. **(C,D)** The local structure of the mutation site in wild-type **(C)** and mutant **(D)** protein. It shows that the formation of two hydrogen bonds inside Arg-89, Pro-88, and Asn-684 makes the local structure stable enough, while this stability is broken by Cys-89 replacing Arg-89.

The p85β regulatory subunit encoded by *PIK3R2* regulates PI3K activity by modulating the stability, conformation, and localization of the catalytic subunit. The structure of the p85β regulatory subunit consists of an Src homology 3 (SH3) domain followed by a Rho-Gap homology region and two Src homology 2 (SH2) regions, which are connected by a coiled-coil region known as the inter-SH2 region (i-SH2) to mediate binding of the p85β regulatory subunit to the catalytic subunit (Fruman et al., 2017; Dornan and Burke, 2018; Vallejo-Diaz et al., 2019; Figure 3A). According to the information from the UniProt database^6^, the p.Arg89Cys mutation is located near the SH3 domain (Figure 3B). In the primary structure of the wild-type p85β regulatory subunit, three amino acids, Arg89, Pro88, and Asn684, form two hydrogen bonds with each other, stabilizing the local structure (Figure 3C). However, in the structure of the p.Arg89Cys variant, the hydrogen bond between Arg89 and Pro88 is broken, leading to instability of the local structure (Figure 3D). We next predicted the effect of this mutation on protein function using the online tool mCSM (Pires et al., 2014) and found that p.Arg89Cys reduces the stability of the protein structure (ΔΔG = −1.374 kcal/mol). In addition, the substitution of positively charged Arg by neutrally charged Cys may cause changes in the local physical and chemical properties of the protein. Therefore, alterations in the structure may further cause changes in the function of the protein.

#### Mutation Frequency and Type Analysis of *PIK3R2*

**FIGURE 4:**
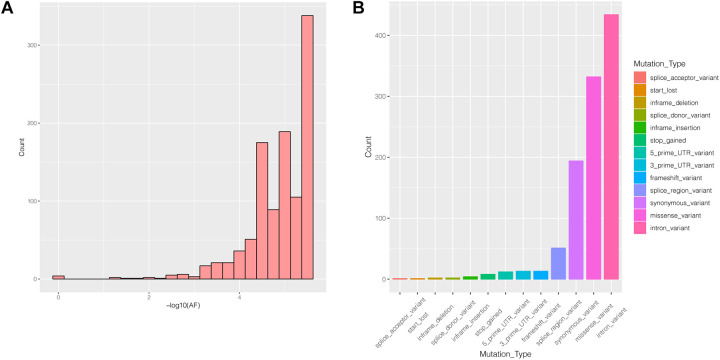
The characters of mutations in the *PIK3R2* gene recorded in gnomAD database. **(A)** The allele frequency (AF) distribution of *PIK3R2* mutations, *x*-axis means the AF values, and we have converted the original value to –log10, and *y*-axis means the number of the mutations. It shows *PIK3R2* is highly conserved as most mutations detected in the gene are rare mutations (MAF < 0.001). **(B)** The annotation information of *PIK3R2* mutations, there are 13 types of annotated mutations in total, and the mutations with the highest frequency are the intron variants (40.67%).

Many genes that are critical for multiple biological processes are conserved in evolution to ensure that they function stably. The function of highly conserved genes is more likely to be affected by mutations. To understand the conservation of *PIK3R2*, we analyzed public data in the gnomAD database and found that most mutations in *PIK3R2* are rare (MAF < 0.001); the most common type of mutation is the intron variant, accounting for more than 40% of all mutation types (Figures 4A,B). Thus, it can be indicated that *PIK3R2* is a highly conserved gene; therefore, mutations in this gene probably affect its physiological function, resulting in the initiation and development of diseases. This has been confirmed by the *PIK3R2* variants causing MPPH1 (Riviere et al., 2012).

#### Relationship Between *PIK3R2* and Known TLE-Related Genes

**FIGURE 5:**
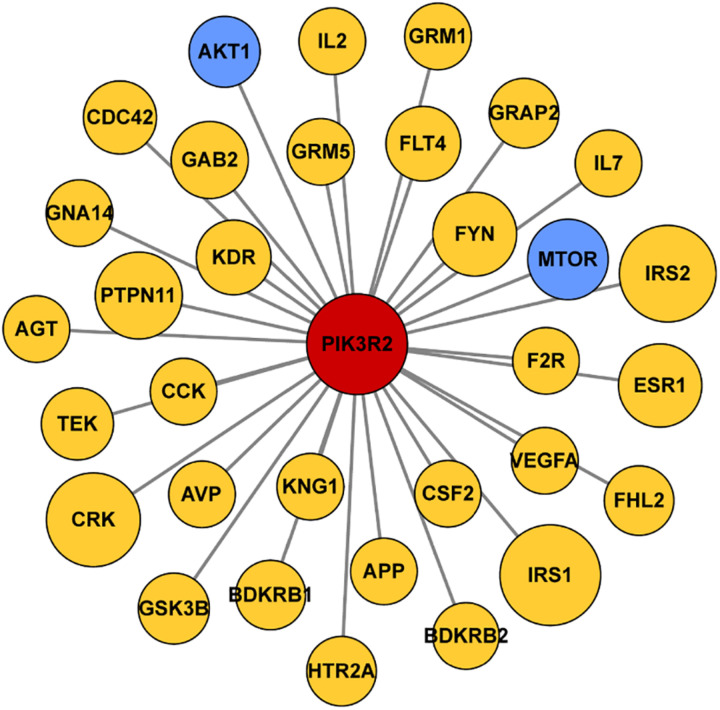
Protein–protein interaction (PPI) network of *PIK3R2* and TLE-related genes. It shows that 32 TLE-related genes interact with *PIK3R2*, based on the information on STRING Database. Two genes in blue color are *AKT1* and *MTOR*, which are components of the PI3K/Akt/mTOR pathway, showing the important role of this pathway in TLE. The area of the circles is proportional to the combined score between *PIK3R2* and TLE-related genes.

Since accumulating evidence has revealed that *PIK3R2* may be involved in epilepsy pathogenesis, we speculate that *PIK3R2* may interact with other epilepsy-related genes. We next searched the DisGeNET platform (see text footnote 4) for TLE-related genes and obtained a gene set containing 354 components. The STRING database (see text footnote 5) was used to construct a protein–protein interaction (PPI) network between the protein products of *PIK3R2* and the TLE-related genes, as shown in Figure 5. The protein products of 32 TLE-related genes from DisGeNET were found to interact with PIK3R2, suggesting that these TLE-related genes are closely related to *PIK3R2*. Furthermore, two genes in the PI3K/Akt/mTOR pathway, *AKT1*, and *MTOR*, are contained in this TLE-related network, showing the importance of this pathway in TLE. Therefore, we infer that *PIK3R2* may play an important role in TLE through extensive interaction with other TLE-related genes.

### Functional Assay

**FIGURE 6:**
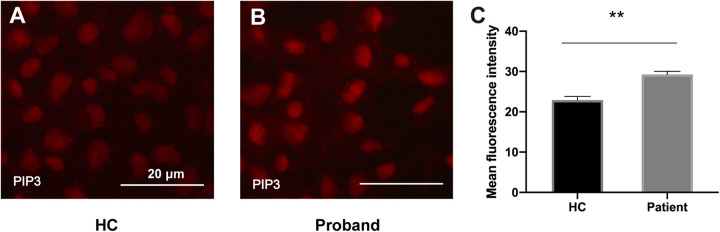
PIP3 expression levels in iPSCs derived from the proband and a healthy control. **(A,B)** Immunofluorescence staining of PIP3 in iPSCs derived from the proband **(A)** and the healthy control **(B)**. Images were taken with the same exposure time. Scale bar corresponds to 20 μm. **(C)** Elevated PIP3 expression is evident in iPSCs derived from the proband. The average PIP3 level among the cells is based on the mean fluorescence intensity, which is calculated with ImageJ software. A two-tailed *t*-test was used to compare the mean fluorescence intensity. ** indicates a significant statistical difference (*p* < 0.01). Error bar indicates SEM.

To assess the functional impact of the *PIK3R2* mutation on the activity of PI3K, we generated an induced pluripotent stem cell (iPSC) line from PBMCs of the proband. As we described previously (Zhang et al., 2020), PBMCs from the proband containing the *PIK3R2* mutation were reprogrammed by a non-integrating vector system with human OKSM transcription factors to induce an iPSC line. Class IA PI3K is a heterodimeric enzyme that converts phosphatidylinositol 4,5-bisphosphate (PIP2) to phosphatidylinositol 3,4,5-trisphosphate (PIP3); hence, the activity of PI3K can be reflected by detecting the level of PIP3 in cells. The iPSCs were used to perform immunofluorescence imaging of PIP3. Compared to iPSCs induced from PBMCs of a healthy control, expression of PIP3 was significantly elevated in iPSCs of the proband, indicating that the activity of PI3K was enhanced (Figures 6A–C). The results suggest that the *PIK3R2* mutation (c.265C > T; p.Arg89Cys) may be a “gain of function” mutation leading to overactivation of PI3K.

### Expression of *PIK3R2* in Patients With Temporal Lobe Epilepsy (TLE)

**FIGURE 7:**
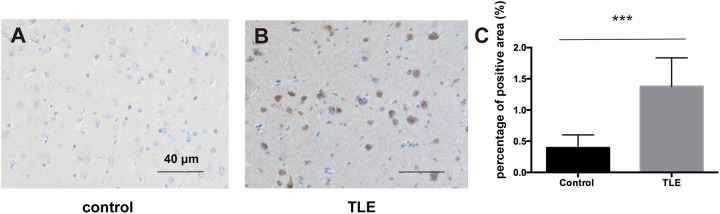
p85β expression levels in the temporal lobe cortex of patients with TLE and controls. **(A)** The expression of p85β is negative in temporal lobe cortex tissues of controls. **(B)** The expression of p85β is significantly positive in the temporal lobe cortex tissues of TLE patients. Scale bar corresponds to 40 μm. **(C)** The statistical results of the percentage of the positive area of p85β in temporal lobe cortex of patients with TLE and controls. Elevated expression of p85β is detected in TLE patients. Mann–Whitney test was used to compare the percentage of the positive area for the non-normal distributions of the data. *** indicates a significant statistical difference (*p* < 0.001). Error bar indicates interquartile range.

Based on the above results, we explored the expression level of *PIK3R2* in refractory TLE patients. We used immunohistochemistry to assess the expression of p85β, the protein product of *PIK3R2*, in temporal lobe tissues of 66 refractory TLE patients compared to tissues of 40 non-epileptic patients with cerebral hemorrhage after surgery as a control. We found significantly elevated levels of p85β staining in patients with TLE compared with control patients (Figures 7A–C). This finding is related to the high proportion of FCD pathological manifestations in the temporal lobe of these TLE patients (unpublished data). The occurrence of FCD is mainly associated with hyperactivation of the PI3K/Akt/mTOR pathway, which may be related to the occurrence of TLE in these patients.

## Discussion

To date, the key genes involved in the initiation and development of FMTLE have not been identified. We identified a missense mutation in the *PIK3R2* gene (NM_005027.4: c.265C > T; NP_005018.2: p.Arg89Cys) for the first time in a family of mTLE. It is also the first time for this mutation to be reported as a potential pathogenic variant. The proband and her youngest sister were patients with mTLE, and their mother was a suspected epilepsy patient. The mutation of *PIK3R2* was detected in all three of them but not in other biological family members. The patients in this family did not show abnormalities in brain structure development, such as polymicrogyria or pachygyria. It should be explained that the symptoms of the mother are milder than the two daughters, we speculate that it was caused by incomplete penetrance of the variant. Since the penetrance of a variant is affected by other genes, epigenetics, and environmental factors, the penetrance of a variant in different individuals could be different. Similar findings have been described by Ishida et al. In their study, three families with familial epilepsy were detected to carry mutations of *DEPDC5* (another component of PI3K/Akt/mTOR pathway). However, the severity of clinical symptoms varies among these patients carrying the same mutation in their respective families, with some even having no clinical symptoms (Ishida et al., 2013). This study indicates that incomplete penetrance is present in familial epilepsy and is not rare.

The *PIK3R2* gene encodes the p85β regulatory subunit of Class IA PI3K. PI3Ks are a conserved family of lipid kinases that phosphorylate the inositol 3′-OH groups of membrane phosphoinositides (PIs). Class I PI3K, consisting of Class IA and IB, which converts phosphatidylinositol 4,5-bisphosphate (PIP2) to phosphatidylinositol 3,4,5-trisphosphate (PIP3) to activate many downstream signaling proteins, is the most well studied of the three classes of PI3K (Dornan and Burke, 2018). It plays an important role in cell proliferation, growth, metabolism, migration, and secretion (Fruman et al., 2017; Dornan and Burke, 2018; Hillmann and Fabbro, 2019; Vallejo-Diaz et al., 2019).

By analyzing data from the gnomAD database, we found that most mutations in *PIK3R2* are rare, indicating that *PIK3R2* is highly conserved; therefore, mutations in this gene may cause diseases. The prediction of protein function shows that the mutation we report herein may lead to a decrease in local structural stability and a change in the physical and chemical properties of the protein, suggesting that the mutation may be a pathogenic variant. To date, *PIK3R2* mutations have been identified in patients with several epilepsy-related diseases, mainly MPPH and BPP, two developmental brain disorders. The most common mutation site of *PIK3R2* in the above diseases is c.1117G > A (p.Gly373Arg) (Riviere et al., 2012; Mirzaa et al., 2015). Another two mutation sites, c.1202T > C (p.Leu401Pro) and c.1669G > C (p.Asp557His), have also been detected in MPPH patients (Nakamura et al., 2014; Terrone et al., 2016). Besides, one mutation site, c.1126A > G (Lys376Glu), was detected in a BPP patient (Mirzaa et al., 2015). In a published summary of 977 epilepsy-related genes found in three databases and recently published papers, *PIK3R2* is classified as a neurodevelopmental epilepsy gene associated with brain developmental malformations and epilepsy (Wang et al., 2017). In this study, it is the first time for the *PIK3R2* mutation (c.265C > T; p.Arg89Cys) to be identified in epilepsy patients and to be found as the potential driver of FMTLE. Furthermore, mutations in other genes in the PI3K/Akt/mTOR pathway, including *AKT3*, *PIK3CA*, and *DEPDC5*, may also cause epilepsy (Riviere et al., 2012; Ishida et al., 2013; Nakamura et al., 2014). Taken together, the mutations in *PIK3R2* and its related signaling pathway are highly associated with epilepsy, suggesting that *PIK3R2* may play an important role in the pathogenesis of epilepsy.

It is worth noting that although these MPPH, BPP, and TLE cases are all caused by *PIK3R2* mutations, their phenotypes are not identical. We speculate that there are two possible reasons for this phenomenon. First, different loci of the variants may lead to different phenotypes. Most pathogenic variants in *PIK3R2* of MPPH and BPP are located in the SH2-1 domain, while the variant we report here is located near the SH3 domain. Second, the function of a gene is affected by multiple factors, including posttranslational modification, gene-gene interactions, environment, etc. Therefore, mutations in the same gene could lead to distinct outcomes in different individuals. A piece of supporting evidence is that a pathogenic variant of *PIK3R2* (c.1681 A > G; p.Asn561Asp) was found to cause endometrial cancer (Cheung et al., 2011), which indicate that mutations in the same gene could result in quite distinct phenotypes.

We used PBMCs derived from the proband and a healthy control to establish iPSC lines as ideal cell tools to perform functional assays of the *PIK3R2* mutation since the mutation is stably harbored in iPSCs from the proband (Zhang et al., 2020). Through cellular immunofluorescence staining, we confirmed that the activity of PI3K in iPSCs derived from the proband with the *PIK3R2* mutation was significantly higher than that in iPSCs from the healthy control. The PPI network of *PIK3R2* and TLE-related genes also shows extensive interactions between protein products of *PIK3R2* and a large number of TLE-related genes, including two key genes in the PI3K/Akt/mTOR pathway, *AKT1* and *MTOR*, suggesting that *PIK3R2* and its related signal pathway may be involved in the pathogenesis of TLE.

In this study, we found that the expression level of *PIK3R2* in the resected temporal lobe of TLE patients was significantly higher than that in the control group. Furthermore, a study on 307 refractory TLE surgery pathologic specimens showed that up to nearly 80% of the cases demonstrated FCD (Gales et al., 2017). Similarly, in our research, most of the temporal lobe tissues of the patients in this TLE cohort had FCD pathological changes, which further suggests that *PIK3R2* plays an important role in TLE and FCD. It is known that excessive activation of the PI3K/Akt/mTOR pathway is a potential cause of FCD, a common pathological manifestation of refractory TLE. It is obvious that FCD is also a developmental brain disorder, which is consistent with previous reports on the pathogenicity of *PIK3R2* gene mutations being mainly associated with brain developmental disorders. Combined with the findings of the FMTLE family in this study, these findings further prove the close relationship between *PIK3R2* and TLE and suggest that the *PIK3R2* mutation may play a pathogenic role in FMTLE.

Overall, our findings suggest that *PIK3R2* plays a pivotal role in the pathogenesis of TLE. We report a mutation in *PIK3R2* functioning unexpectedly as a potential pathogenic variant in an FMTLE family. Although *PIK3R2* mutations are known to be responsible for epilepsy-related brain developmental disorders, including MPPH and BPP, there are no reports on the relationship between *PIK3R2* mutations and FMTLE. More importantly, no pathogenic variant has been identified in FMTLE patients to date, which makes our findings important. However, this *PIK3R2* mutation needs to be verified in a larger population of FMTLE patients and further explored regarding its possible pathogenic mechanism.

## Data Availability Statement

The original contributions presented in the study are included in the article/Supplementary Material, further inquiries can be directed to the corresponding author/s.

## Ethics Statement

The studies involving human participants were reviewed and approved by the Medical Ethics Committee of Renji Hospital Affiliated with Shanghai Jiao Tong University School of Medicine. The patients/participants provided their written informed consent to participate in this study. Written informed consent was obtained from the individual(s) for the publication of any potentially identifiable images or data included in this article.

## Author Contributions

YW contributed to functional assay, bioinformatics analysis—PPI network, prediction of functional impairment of proteins, and original draft preparation. JP contributed to immunohistochemistry of TLE and control patients. SB and HH contributed to bioinformatics analysis—structure modeling, mutation frequency, and type analysis of *PIK3R2*. HY contributed to statistical analysis. CF contributed to clinical data collection. YH and YG contributed to conception and design of the research, review and editing of the manuscript, project administration, and funding acquisition. All authors contributed to the article and approved the submitted version.

## Conflict of Interest

The authors declare that the research was conducted in the absence of any commercial or financial relationships that could be construed as a potential conflict of interest.

## Footnotes

1 http://grch37.genepipe.ncgm.sinica.edu.tw/primerz/beginDesign.do

2 https://zhanglab.ccmb.med.umich.edu/I-TASSER/

3 http://www.pymol.org

4 https://www.disgenet.org

5 https://string-db.org

6 https://www.uniprot.org
