# Supplementary material for: A PIK3R2 Mutation in Familial Temporal Lobe Epilepsy as a Possible Pathogenic Variant
Source: Front Genet. 2021 May 10;12:596709. doi: 10.3389/fgene.2021.596709 (PMC8141861; doi:10.3389/fgene.2021.596709)
Supplement: Supplementary file 1 [file Table_1.DOCX]

Supplementary Material

# Supplementary Table 1.

# 511 selected epilepsy-related genes in the panel for next-generation sequencing of the DNA derived from the proband.

| ABAT | CHRNA2 | GABRA4 | IER3IP1 | MAP2 | PIR | SLC35A3 |
| --- | --- | --- | --- | --- | --- | --- |
| ABCC2 | CHRNA4 | GABRA5 | IL6ST | MBD5 | PKHD1 | SLC35C1 |
| ABCC8 | CHRNA5 | GABRA6 | IMPA2 | MBTPS2 | PLCB1 | SLC46A1 |
| ACADM | CHRNA7 | GABRB1 | INA | ME2 | PNKD | SLC4A3 |
| ACO2 | CHRNB2 | GABRB2 | IQSEC2 | MECP2 | PNKP | SLC52A2 |
| ACOX1 | CLCN2 | GABRB3 | JRK | MED17 | PNPO | SLC6A11 |
| ACP1 | CLCN4 | GABRD | JUN | MEF2C | POLG | SLC6A13 |
| ACSF3 | CLCNKB | GABRE | KARS | MFSD8 | POLG2 | SLC6A19 |
| ACTA2 | CLEC18A | GABRG1 | KCNA1 | MIB1 | POMT1 | SLC6A8 |
| ACTB | CLIC2 | GABRG2 | KCNA2 | MMADHC | POMT2 | SLC9A3 |
| ACY1 | CLN3 | GABRG3 | KCNA6 | MOCS1 | PPP1R3C | SLC9A6 |
| ADAR | CLN5 | GABRP | KCNAB1 | MOCS2 | PPT1 | SLC9A9 |
| ADCK3 | CLN6 | GABRQ | KCNAB2 | MOG | PRF1 | SMARCA2 |
| ADK | CLN8 | GABRR1 | KCNAB3 | MSN | PRICKLE1 | SMARCA4 |
| ADSL | CNP | GABRR2 | KCNB1 | MTHFR | PRICKLE2 | SMC1A |
| AFG3L2 | CNR1 | GABRR3 | KCNC1 | MTMR9 | PRIMA1 | SNIP1 |
| AGTR2 | CNTF | GAD1 | KCNC2 | MTOR | PRNP | SNX27 |
| AKT3 | CNTN2 | GAMT | KCNC4 | MTR | PRODH | SOBP |
| ALDH4A1 | CNTNAP2 | GATM | KCND1 | NAGA | PRRT2 | SPAST |
| ALDH5A1 | COG6 | GBA | KCND2 | NALCN | PSAP | SPATA5 |
| ALDH7A1 | COG8 | GCDH | KCNE1L | NDE1 | PSAT1 | SPR |
| ALG13 | COL4A1 | GCH1 | KCNE2 | NDP | PSEN1 | SPTAN1 |
| AMACR | COQ2 | GCM2 | KCNF1 | NDUFV1 | PTEN | SRGAP2 |
| AMT | COQ9 | GCSH | KCNG1 | NF1 | PTH | SRPX2 |
| ANK3 | CPA6 | GJA1 | KCNG4 | NGLY1 | PTS | ST3GAL3 |
| ANKRD11 | CPS1 | GJD2 | KCNH2 | NHLRC1 | QDPR | ST3GAL5 |
| AP4E1 | CPT2 | GLB1 | KCNH3 | NIPA2 | RAB39B | STRADA |
| ARG1 | CSTB | GLDC | KCNH8 | NPRL2 | RANBP2 | STXBP1 |
| ARHGEF15 | CTDP1 | GLRA1 | KCNJ1 | NPRL3 | RBFOX1 | SUOX |
| ARHGEF9 | CTSD | GLRB | KCNJ10 | NPY | RBPJ | SV2A |
| ARSA | CUL4B | GLUD1 | KCNJ11 | NRXN1 | RELN | SYN1 |
| ARX | CYP4F11 | GM2A | KCNJ2 | NSDHL | RFT1 | SYNGAP1 |
| ASAH1 | D2HGDH | GOSR2 | KCNJ3 | NSF | RHAG | SYP |
| ASIC1 | DCX | GPHN | KCNJ5 | NTRK1 | RHOA | SYT11 |
| ASPA | DDC | GPR56 | KCNJ6 | NTRK2 | RMND1 | SZT2 |
| ASPM | DEAF1 | GPR98 | KCNJ9 | OPA1 | RNASEH2A | TBC1D24 |
| ASS1 | DEPDC5 | GRIA1 | KCNK1 | OPHN1 | RNF213 | TBCE |
| ATIC | DGKD | GRIA2 | KCNK3 | OPRM1 | ROGDI | TBX1 |
| ATN1 | DHFR | GRIA3 | KCNK7 | OTX2 | RPS6KA3 | TCF4 |
| ATP1A2 | DIAPH1 | GRIA4 | KCNK9 | PAFAH1B1 | SCARB2 | TEAD1 |
| ATP1A3 | DIAPH3 | GRIK1 | KCNMA1 | PAH | SCN1A | TICAM1 |
| ATP2A2 | DLX2 | GRIK2 | KCNMB2 | PALLD | SCN1B | TK2 |
| ATP6AP2 | DLX5 | GRIK3 | KCNMB3 | PARK2 | SCN2A | TLR3 |
| ATP6V0A2 | DNM1 | GRIK4 | KCNMB4 | PC | SCN2B | TMEM67 |
| ATP7A | DPM1 | GRIK5 | KCNN2 | PCDH19 | SCN3A | TNF |
| AUH | DPYS | GRIN1 | KCNQ1 | PDCD10 | SCN3B | TPP1 |
| BCKDK | DYNC1H1 | GRIN2A | KCNQ2 | PDHA1 | SCN4A | TPRXL |
| BDNF | DYRK1A | GRIN2B | KCNQ3 | PDHX | SCN5A | TREX1 |
| BRAT1 | EEF1A2 | GRIN2C | KCNQ4 | PDYN | SCN7A | TRMT44 |
| BRD2 | EFHC1 | GRIN2D | KCNQ5 | PEX1 | SCN8A | TRPM6 |
| BTD | EFHC2 | GRIN3A | KCNS2 | PEX10 | SCN9A | TSC1 |
| C10ORF2 | EGF | GRIN3B | KCNS3 | PEX12 | SDHA | TSC2 |
| CACNA1A | EHMT1 | GRM1 | KCNT1 | PEX13 | SEPSECS | TSEN2 |
| CACNA1E | EIF2AK3 | GRN | KCNV1 | PEX14 | SERPINI1 | TSEN34 |
| CACNA1G | ELOVL4 | GTDC2 | KCNV2 | PEX16 | SETD2 | TSEN54 |
| CACNA1H | ELP4 | HAX1 | KCTD7 | PEX19 | SEZ6 | TSPO |
| CACNA2D2 | EMX2 | HCCS | KDM5C | PEX2 | SHANK3 | TUBA1A |
| CACNG2 | EN2 | HCFC1 | KIF1A | PEX26 | SLC12A1 | TUBA8 |
| CALHM1 | EPM2A | HCN1 | KLK1 | PEX3 | SLC12A5 | TUBB2A |
| CASK | EPM2AIP1 | HCN2 | KRIT1 | PEX5 | SLC12A6 | TUBB2B |
| CASP9 | FADD | HCN4 | L1CAM | PEX6 | SLC13A5 | TUBGCP6 |
| CASR | FARS2 | HDAC4 | LAMA2 | PFKL | SLC16A1 | U2AF1 |
| CBS | FKTN | HEPACAM | LAMB1 | PFKM | SLC17A5 | UBC |
| CCDC88C | FLNA | HEXA | LBR | PHF6 | SLC19A3 | UBE2A |
| CCL3 | FOLR1 | HEXB | LGI1 | PHGDH | SLC1A1 | UBE3A |
| CCL4 | FOS | HLCS | LGI2 | PHOX2A | SLC1A2 | VAMP2 |
| CCM2 | FOXG1 | HNRNPU | LGI4 | PIGA | SLC1A3 | VLDLR |
| CDK5 | FUCA1 | HOXA1 | LIAS | PIGL | SLC20A2 | VPS13A |
| CDKL5 | GABBR1 | HPD | LIFR | PIGN | SLC25A12 | WARS |
| CDYL | GABBR2 | HSD17B10 | LMBRD1 | PIGO | SLC25A13 | WDR45 |
| CENPJ | GABRA1 | HTR1A | LMNB2 | PIGV | SLC25A22 | WDR62 |
| CHD2 | GABRA2 | IDH2 | MANBA | PIK3CA | SLC2A1 | ZEB2 |
| CHRFAM7A | GABRA3 | IDS | MAOB | PIK3R2 | SLC35A2 | ZNF238 |
