# Supplementary material for: A PIK3R2 Mutation in Familial Temporal Lobe Epilepsy as a Possible Pathogenic Variant
Source: Front Genet. 2021 May 10;12:596709. doi: 10.3389/fgene.2021.596709 (PMC8141861; doi:10.3389/fgene.2021.596709)
Supplement: Supplementary file 2 [file Table_2.DOCX]

Supplementary Material

# Supplementary Table 2.

# The CADD scores of *PIK3R2* variant (c.265C>T; p.Arg89Cys) we report here and other pathogenic variants of *PIK3R2* recorded in ClinVar Database to date.

| Variant locus | CADD score | Related disease or disorder |
| --- | --- | --- |
| c.265 C>T (p.Arg89Cys) | 24.3 | FMTLE |
| c.988 T>G (p.Trp330Gly) | 30 | MPPH |
| c.1056 C>G (p.Phe352Leu) | 23.5 | MPPH |
| c.1117 G>A (p.Gly373Arg) | 24.6 | MPPH |
| c.1153 G>A (p.Gly385Arg) | 24.2 | MPPH |
| c.1202 T>C (p.Leu401Pro) | 24.1 | MPPH |
| c.1126 A>G (p.Lys376Glu) | 24.2 | BPP |
| c.1681 A>G (p.Asn561Asp) | 26.8 | Endometrial cancer |
